# Serum miR‐195‐5p is upregulated in gestational diabetes mellitus

**DOI:** 10.1002/jcla.23325

**Published:** 2020-04-17

**Authors:** Jianping Wang, Yuanyuan Pan, Fen Dai, Fan Wang, Haifan Qiu, Xianping Huang

**Affiliations:** ^1^ Department of Obstetrics and Gynecology The second Affiliated Hospital of Wenzhou Medical University Zhejiang China

**Keywords:** correlation, gestational diabetes mellitus, miR‐195‐5p, ROC curve

## Abstract

**Background:**

Gestational diabetes mellitus (GDM) is defined as varying degrees of glucose intolerance with an onset or first recognition during pregnancy in women without previously diagnosed diabetes. Accumulating evidence indicates that miRNAs exert crucial roles in the pathogenesis and development of diabetes, including GDM. In the present study, we aimed to determine the clinical performance of miR‐195‐5p in GDM.

**Methods:**

First, the miR‐195‐5p expressions in serum samples from healthy pregnant women and women with GDM at 25 weeks pregnancy were detected using real‐time polymerase chain reaction (RT‐qPCR). Then, receive characteristic (ROC) curve was used to determine the diagnostic value of miR‐195‐5p in GDM. Finally, the correlation analysis of miR‐195‐5p expression with related clinicopathological factors was carried out to determine the clinical value of miR‐195‐5p in GDM.

**Results:**

In this study, we found that miR‐195‐5p expression was significantly increased in serum samples from GDM patients as compared with that in healthy pregnancies. Furthermore, miR‐195‐5p might be a putative biomarker for GDM diagnosis with an area under the curve (AUC) of 0.8451; the cutoff value was 1.598, sensitivity was 73.69%, specificity was 96.85%, accuracy was 81.26%, and Youden index was 70.54%. Expression of miR‐195‐5p was positively associated with fasting plasma glucose, one‐hour plasma glucose, and two‐hour plasma glucose.

**Conclusion:**

miR‐195‐5p might function as a putative diagnostic biomarker for GDM and contribute to identifying at‐risk mothers in pregnancy.

## 1 INTRODUCTION

Gestational diabetes mellitus (GDM) is defined as any degree of glucose intolerance among pregnant women without a previous history of diabetes.^1^ GDM affects about 1.1%‐14.3% of pregnant women with about 35%‐70% recurrence risk.^2^, ^3^ Furthermore, GDM may also result in adverse clinical effects, including pre‐eclampsia, birth defects, and cesarean sections.^4^ Hence, it is necessary to find an easy way to identify those at‐risk mothers in pregnancy.

miRNAs are a group of small, non‐coding RNAs (~22 nucleotides in length) that could regulate gene expression by degradation or restraining translation of messenger RNA transcription. To our knowledge, aberrant miRNA expression has been demonstrated in various diseases, including diabetes and GDM.^5^, ^6^ Meanwhile, several miRNAs have been reported to be related to insulin secretion and insulin resistance, which are key factors in diabetes diagnosis and treatment.^7^, ^8^ A previous study noted that miR‐195‐5p was upregulated in GDM patients.^9^ However, the relationship between miR‐195‐5p and GDM remains unclear.

In the present study, we determined the expression level of miR‐195‐5p in GDM patients. Additionally, we tested the clinical roles of miR‐195‐5p in GDM to contribute to clinical diagnosis and treatment.

## 2 METHODS

### 2.1 Patient samples

Serum samples from 24 weeks ~ 28 weeks pregnancy were obtained from 204 patients from the Second Affiliated Hospital of Wenzhou Medical University, including 102 GDM patients and 102 normal pregnant women collected in red tiger‐top gel separator tubes (Thermo Fisher Scientific). GDM was diagnosed if the fasting plasma glucose level was ≥5.1 mmol/L. GDM was excluded if the fasting plasma glucose was ≤4.4 mmol/L. Pregnancies with fasting glucose between 4.4 and 5.1 mmol/L underwent a 75 g oral glucose tolerance test (OGTT). Under this circumstance, a diagnosis of GDM was verified when at least one glucose value was elevated (fasting glucose ≥5.1 mmol/L, 1‐hour OGTT ≥10.0 mmol/L, or 2‐h OGTT ≥8.5 mmol/L). Patients were excluded if they were diagnosed with cardiovascular diseases, cancers or any other major illness, and pregnancies with GDM who did not fulfill any of the exclusion criteria were recruited in the current study.

Participants fasted overnight before tests under standardized conditions. Fasting blood samples were taken from participants and collected in evacuated silicon‐coated tubes containing gel for the separation of sera from the blood clot. After the coagulation of the blood, serum samples were separated by centrifugation at 10 000 g and kept at −80°C until use. Basic clinicopathological factors in 204 samples are displayed in Table 1. The study was approved by the Ethics Committee of the Second Affiliated Hospital of Wenzhou Medical University, and written informed consent was obtained from all participants.

**Table 1:** The clinicopathological factors of healthy pregnancies and GDM patients

Characteristics	Healthy (N = 102)	GDM (N = 102)	T value	*P* value
Age (y)	29.5 ± 2.8	29.8 ± 3.2	0.7126	.4769
BMI (kg/m^2^)	22.6 ± 3.4	28.3 ± 4.8	9.7867	<.0001
Gestation (wk)	26.8 ± 1.1	27.0 ± 1.6	1.0403	.2994
Fasting plasma glucose (mM)	4.5 ± 0.3	5.3 ± 0.1	25.5500	<.0001
One‐hour plasma glucose (mM)	6.3 ± 0.2	10.2 ± 0.3	109.2428	<.0001
Two‐hour plasma glucose (mM)	5.5 ± 0.4	8.3 ± 0.2	63.2329	<.0001
Fetal birth weight (grams)	3420 ± 268	3430 ± 196	0.3042	.7613

Note

Student's *t* test was used to measure differences between healthy controls and GDM patients.

All the participants meet the following criteria: abstinence from smoking and alcohol at least 4 days before the study, willingness to sign the defined protocol over the whole study period, and continuance of usual medication and insulin administration, if necessary.

### 2.2 Laboratory parameters

Under the guidelines of the German Medical Association,^10^ glucose concentrations were detected using continuous glucose monitoring systems (CGMS), glucometer system Calla Premium, and glucose oxidase strips (Wellion).

### 2.3 Reverse transcription‐quantitative real‐time PCR (RT‐qPCR) assay

Total RNA was extracted from 500 μL serum samples using TRIzol LS reagent (Life Technologies) following the manufacturer's instructions. RNA samples were quantified in a NanoDrop One spectrophotometer (Thermo Fisher Scientific), and the quality and integrity were checked in a 2100 Bioanalyzer (Agilent). cDNA (2 μL) was synthesized using the TaqMan MicroRNA Reverse Transcription kit (Applied Biosystems). Then, the expression of miR‐195‐5p was detected by SYBR Green PCR kit (Qiagen) according to the manufacturer's protocol. The thermal cycling conditions were as follows: 94°C for 3 minutes, 45 cycles of 94°C for 5 seconds, 65°C for 20 seconds, and final extension at 65°C for 5 minutes. U6 functioned as an internal control. The relative expression of miR‐195‐5p was normalized to the level of U6 small nuclear RNA using the 2^−△△^ *^C^* ^t^ method. The sequences were as follows: miR‐195‐5p forward, 5′‐GGAGTGTAGGCCCAATACCAGA‐3′; and reverse 5′‐TGCCACTTAGCAGCACAGAAA‐3′; U6 forward, 5′‐AGAGCCTGTGGTGTCC‐3′; and reverse 5′‐CATCTTCAAAGCACTTCCCT‐3′.

### 2.4 Statistical analysis

All data analyses were performed using the SPSS version 17.0 statistical software and presented as mean ± standard deviation (SD). Receiver operating characteristic curve (ROC) was constructed to determine the clinical diagnostic value of miR‐195‐5p in GDM, followed by the post hoc Tukey's test was used to distinguish the differences between the two groups. The Pearson correlation analysis was used to verify the correlation between miR‐195‐5p and related factors. A *P* value <.05 was considered statistically significant.

## 3 RESULTS

### 3.1 miR‐195‐5p is upregulated in the serum of GDM patients

**Figure 1:**
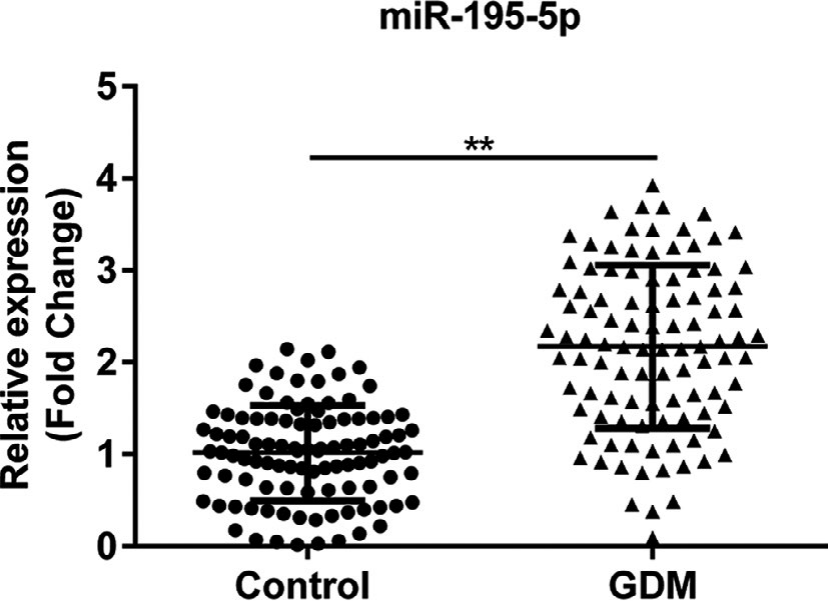
The expression of miR‐195‐5p in the serum of 204 samples from healthy pregnant women and GDM patients. Control, serum samples from healthy pregnant women; GDM, serum samples from GDM patients. **P < .01

As demonstrated in Figure 1, the expression of miR‐195‐5p was significantly increased in the serum of GDM patients as compared to those in healthy pregnant women (^**^ *P* < .01).

### 3.2 Clinicopathological factors of healthy pregnancies and GDM patients

**Figure 2:**
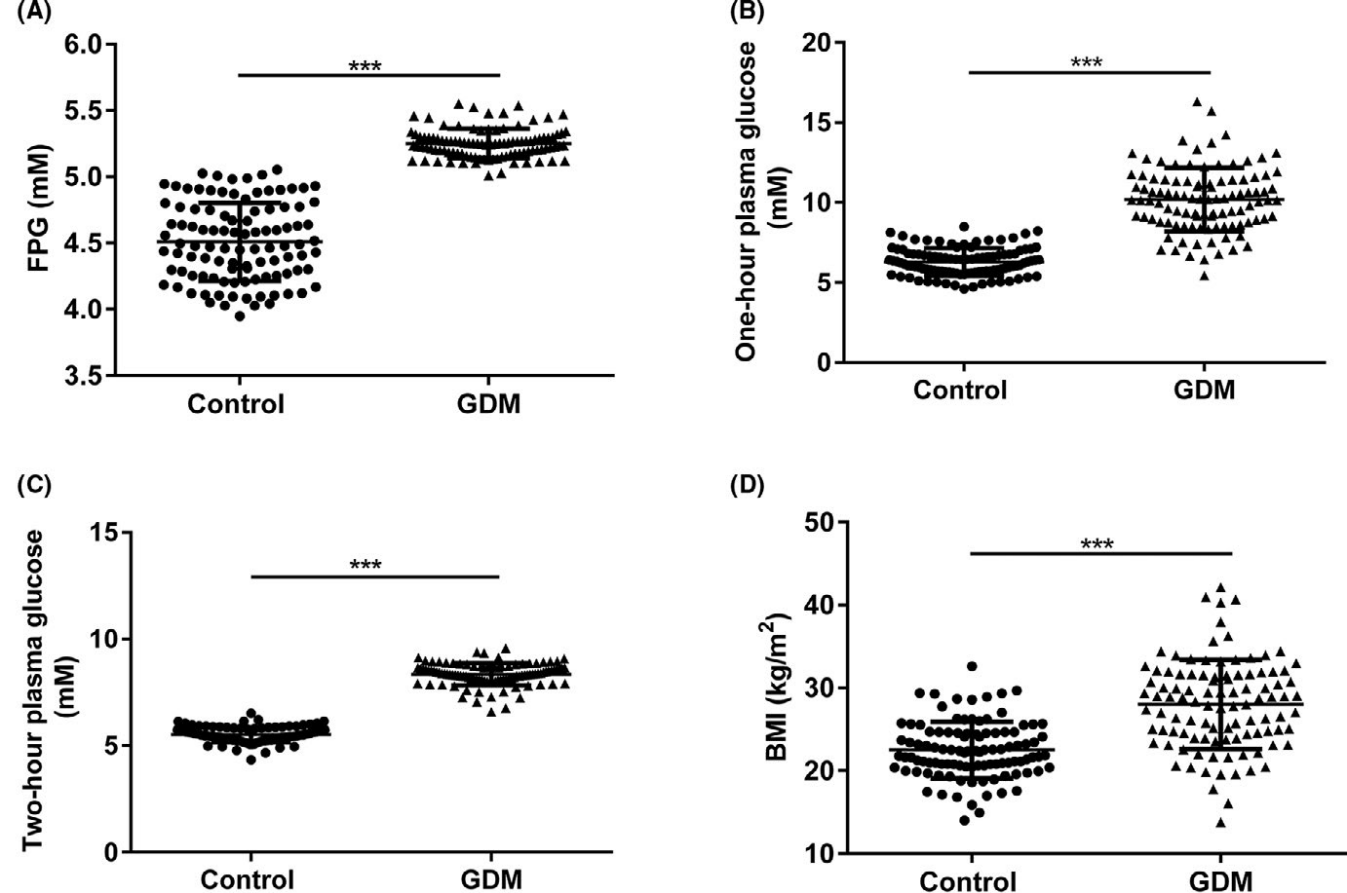
The expressions of fasting plasma glucose, one‐hour plasma glucose, two‐hour plasma glucose, and BMI in the serum of 204 samples from healthy pregnant women and GDM patients. Control, serum samples from healthy pregnant women; GDM, serum samples from GDM patients; FPG, fasting plasma glucose. ^***^ *P* < .0001

As demonstrated in Table 1, there are significant differences in body mass index (BMI), fasting plasma glucose, one‐hour plasma glucose, and two‐hour plasma glucose between the two groups, while there are no significant differences in age, gestational age, and fetal weight. As demonstrated in Figure 2A‐D, fasting plasma glucose, 1‐hour plasma glucose, two‐hour plasma glucose, and BMI were significantly higher in the serum of GDM patients as compared with healthy volunteers (^***^ *P* < .0001).

### 3.3 The diagnosis value of miR‐195‐5p in GDM

**Figure 3:**
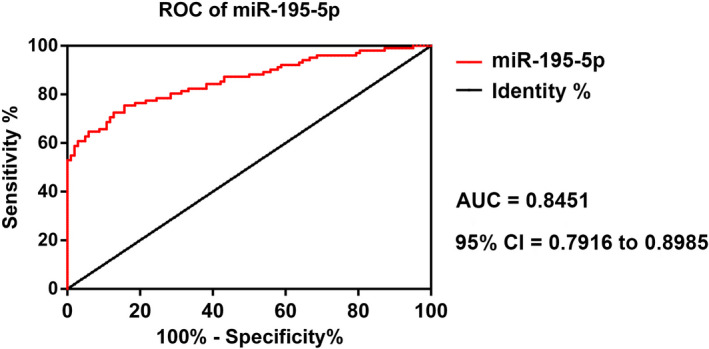
The ROC curve of miR‐195‐5p. ROC, receiver operating characteristics

In the present study, the ROC curve was used to measure the diagnostic value of miR‐195‐5p in GDM. The area under the curve (AUC) value was 0.8451, while the 95% confidence interval ranged from 0.7916 to 0.8985, and the cutoff value was 1.598, sensitivity was 73.69%, specificity was 96.85%, accuracy was 81.26%, and Youden index was 70.54%, which suggested a remarkable performance for miR‐195‐5p in GDM diagnosis (Figure 3 and Table 2).

**Table 2:** The diagnostic value of miR‐195‐5p for GDM

Biomarker	Cutoff value	Sensitivity (%)	Specificity (%)	Accuracy (%)	Youden index (%)	AUC	*P*
miR‐195‐5p	1.598	73.69	96.85	81.26	70.54	0.8451 (0.7916 ~ 0.8985)	<.0001

### 3.4 miR‐195‐5p expression is positively correlated with fasting plasma glucose, one‐hour plasma glucose, two‐hour plasma glucose, and BMI

**Figure 4:**
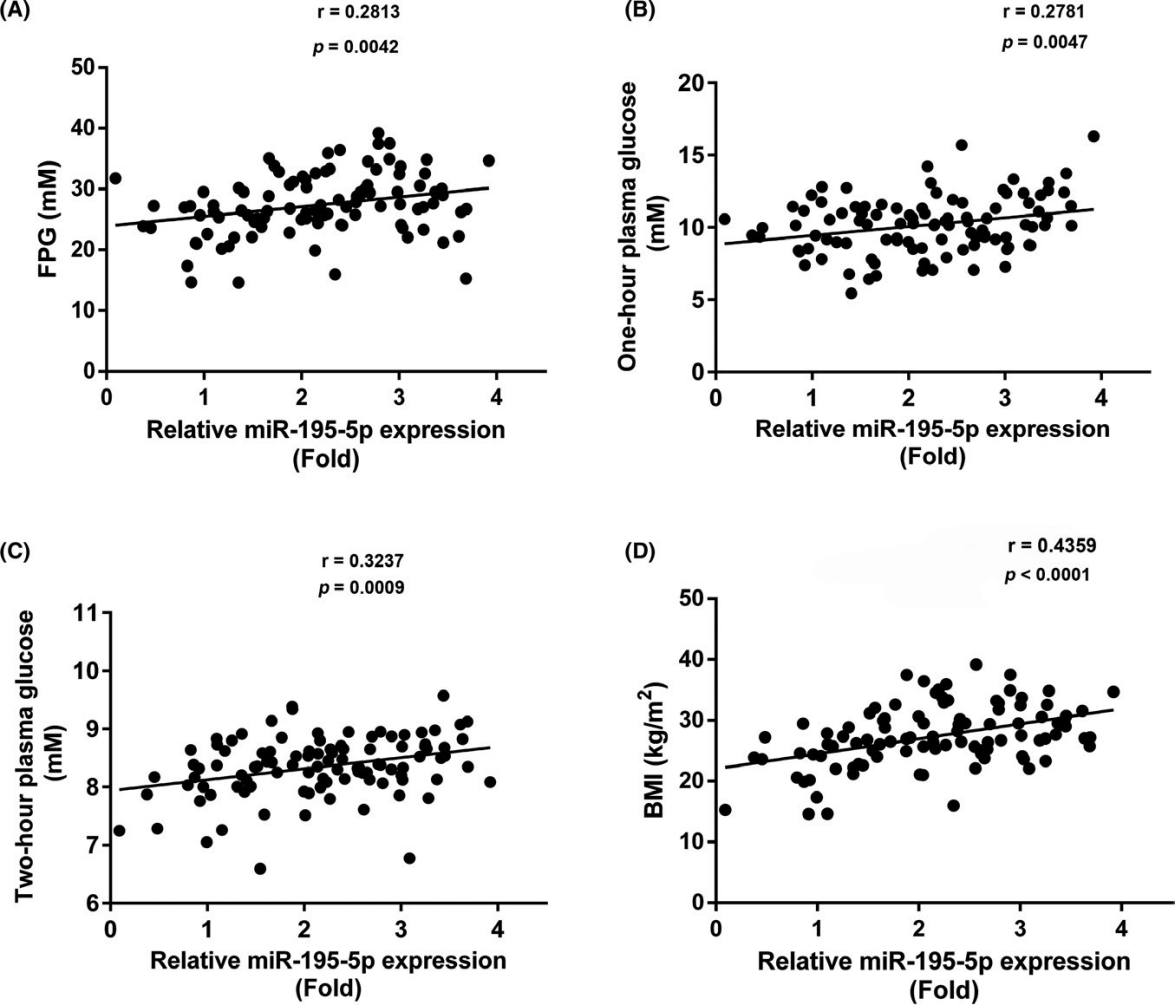
The correlation analysis of miR‐195‐5p expression and fasting plasma glucose, one‐hour plasma glucose, two‐hour plasma glucose, and BMI. FPG, fasting plasma glucose

The expression of miR‐195‐5p was positively correlated with fasting plasma glucose (*r* = .2667, *P* = .0067), one‐hour plasma glucose (*r* = 0.2781, *P* = .0047), two‐hour plasma glucose (*r* = 0.3237, *P* = .0009), and BMI (*r* = 0.4359, *P* < .0001; Figure 4A‐D).

## 4 DISCUSSION

Several studies have reported the crucial role of miRNAs in the pathogenesis and development of metabolic diseases, including GDM. The initial study of the predictive function of miRNAs in diabetes was reported in 2004.^11^ Meanwhile, previous studies also reported that specific miRNAs could also control insulin expression, secretion, and processing.^12^ According to a former study, miR‐195‐5p was also involved in regulating insulin resistance, which was a key contributor in the pathogenesis of GDM.^13^ Meanwhile, a previous study noted that miR‐195‐5p expression was remarkably increased in women with gestational diabetes as compared to healthy volunteers,^9^ which was consistent with our study.

According to the International Association of Diabetes in Pregnancy Group's recommendations, an oral glucose tolerance test is used to diagnose GDM during pregnancy.^14^ miR‐195‐5p, functioning as a diagnostic test, may contribute beneficially to both patients and physicians. Compared to traditional oral glucose tolerance tests,^15^ serum may function as an adjuvant diagnostic method for GDM Yang et al observed that miR‐195 is increased by saturated fatty acids, which aimed to impair insulin signaling and glycogen metabolism in HepG2 cells and increased the risk of metabolic diseases.^16^, ^17^ A previous study also implied the potential diagnostic value of miR‐195‐5p in the diabetes model or related diseases. For instance, miR‐195 expression varied with hyperglycemia, suggesting a potential role of miR‐195 in the pathophysiology of type 2 diabetes.^18^

Obesity may result in insulin resistance and thereby lead to diabetes.^19^ miR‐195‐5p expression was reported to be correlated with the BMI in patients with metabolic syndrome.^20^ Consistent with previous studies, we detected that miR‐195‐5p was positively associated with obesity, fasting glucose, 1‐hour glucose, and 2‐hour glucose, indicating that aberrant expression of miR‐195‐5p might function as a novel diagnostic biomarker in GDM. However, our study does have some limitations. For instance, large sample numbers are necessary to validate the feasibility of using serum miR‐19505p as a diagnostic test for GDM. In the future, we will recruit more volunteers and GDM patients to further confirm our assumptions.
